# Unmasking the Hidden Threat: The Role of Left Ventricular Subendocardial Involvement in Autoimmune Rheumatic Disease

**DOI:** 10.1002/clc.70069

**Published:** 2025-01-01

**Authors:** Danni Wu, Xiao Li, Tianchen Guo, Xiaojin Feng, Xinhao Li, Yining Wang, Wei Chen

**Affiliations:** ^1^ Department of Cardiology Peking Union Medical College Hospital, Chinese Academy of Medical Sciences & Peking Union Medical College Beijing China; ^2^ Department of Radiology Peking Union Medical College Hospital, Chinese Academy of Medical Science & Peking Union Medical College Beijing China

**Keywords:** adverse outcomes, autoimmune rheumatic disease, late gadolinium enhancement, radiomics

## Abstract

**Background:**

Late gadolinium enhancement (LGE) has been found in patients with autoimmune rheumatic disease (ARD). However, the prognostic implications of some specific LGE patterns in ARD patients remain unclear.

**Purpose:**

To investigate the prevalence and prognostic significance of left ventricular (LV) subendocardium‐involved LGE (LGEse) in a cohort of ARD patients.

**Materials and Methods:**

This retrospective study evaluated 176 patients diagnosed with ARD with clinically suspected cardiac involvement between 2018 and 2023. LV LGEse was defined as LGE involving the LV subendocardium that did not correspond to a coronary vascular distribution. The endpoints included a composite of cardiac death, heart failure‐related admission, cardiogenic shock, and appropriate pacemaker or implantable cardioverter‐defibrillator therapy.

**Results:**

Of the 176 consecutive patients, LV LGEse was observed in 22 patients (13%). During a median follow‐up of 776 days (interquartile range, 395–1405 days), 20 patients (11%) experienced a composite endpoint. Compared with those without LV LGEse, the LV LGEse group had a greater proportion of men (64% vs. 14%; *p* < 0.001), lower LV ejection fraction (50% vs. 60%; *p* = 0.001), greater LV end‐diastolic volume index (78 vs. 75; *p* = 0.043), and more adverse outcomes (32% vs. 8%; *p* = 0.005). In the univariable and multivariable Cox regression analyses, the LV LGEse showed independent prognostic value. In the sensitivity analyses, the prognostic difference in terms of LV subendocardial involvement remained.

**Conclusion:**

In our cohort, LV subendocardial involvement, an underrecognized LGE pattern, was observed in 13% of all patients with autoimmune disease and indicated a worse prognosis.

## Graphical abstract

Cardiac magnetic resonance‐detected left ventricular subendocardial late gadolinium enhancement was more strongly associated with adverse outcomes in autoimmune rheumatic disease patients. LV, left ventricular. LGE, late gadolinium enhancement.

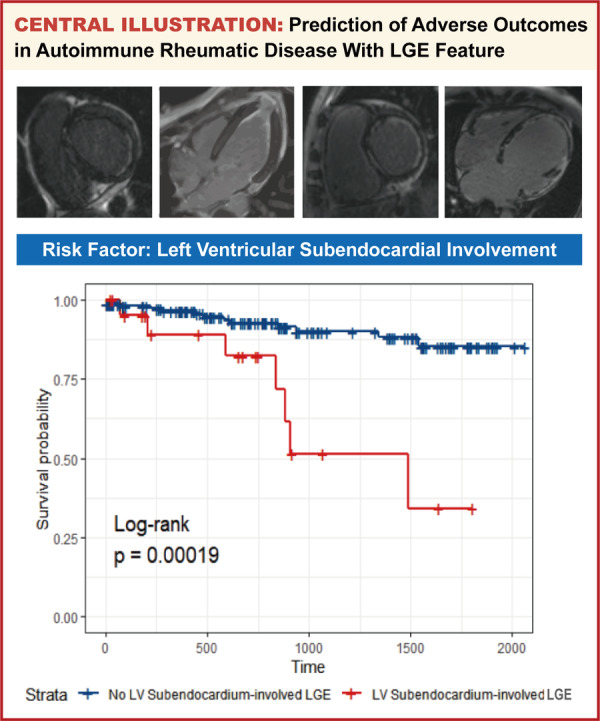

## 1 Introduction

Autoimmune rheumatic diseases (ARDs) encompass a variety of conditions, such as systemic lupus erythematosus (SLE), idiopathic inflammatory myopathies, and systemic vasculitis, all of which can lead to significant cardiac involvement [1, 2]. Cardiac magnetic resonance (CMR) imaging with late gadolinium enhancement (LGE) has become a pivotal tool for detecting myocardial fibrosis and inflammation in these patients [3]. While LGE patterns, such as mid‐wall and epicardial enhancement, are well documented in ARD patients, the clinical significance of left ventricular (LV) subendocardial involvement has been underrecognized and underreported [4, 5, 6, 7, 8, 9]. Emerging evidence suggests that subendocardial LGE (LGEse), often associated with ischemic heart disease, may also be present in ARD patients and could potentially serve as an indicator of adverse cardiac outcomes [10, 11, 12]. However, the prognostic implications of this specific LGE pattern in ARD patients remain unclear. We hypothesize that LV LGEse is associated with a greater incidence of adverse cardiac outcomes in ARD patients. The primary aim of this study was to investigate the prevalence and prognostic significance of LV LGEse in a cohort of ARD patients. Specifically, we sought to determine whether the presence of this LGE pattern is predictive of increased rates of cardiac death, heart failure, and other severe cardiac events.

## 2 Methods

### 2.1 Study Population

We retrospectively evaluated 214 patients who were diagnosed with at least one autoimmune disease with clinically suspected cardiac involvement from January 2018 to April 2023. All patients were assessed by 12‐lead or 24‐h Holter electrocardiography and cardiac magnetic resonance (CMR) imaging within 2 weeks before echocardiography. Autoimmune diseases were diagnosed according to classification criteria, which are detailed in the Supplemental Methods. These patients with clinically suspected cardiac involvement met the CMR indications, including new‐onset or persisting cardiac symptoms (chest pain, chest distress, palpitation, or dyspnea), electrocardiogram (ECG) abnormalities or elevated cardiac troponin I.

**Figure 1:**
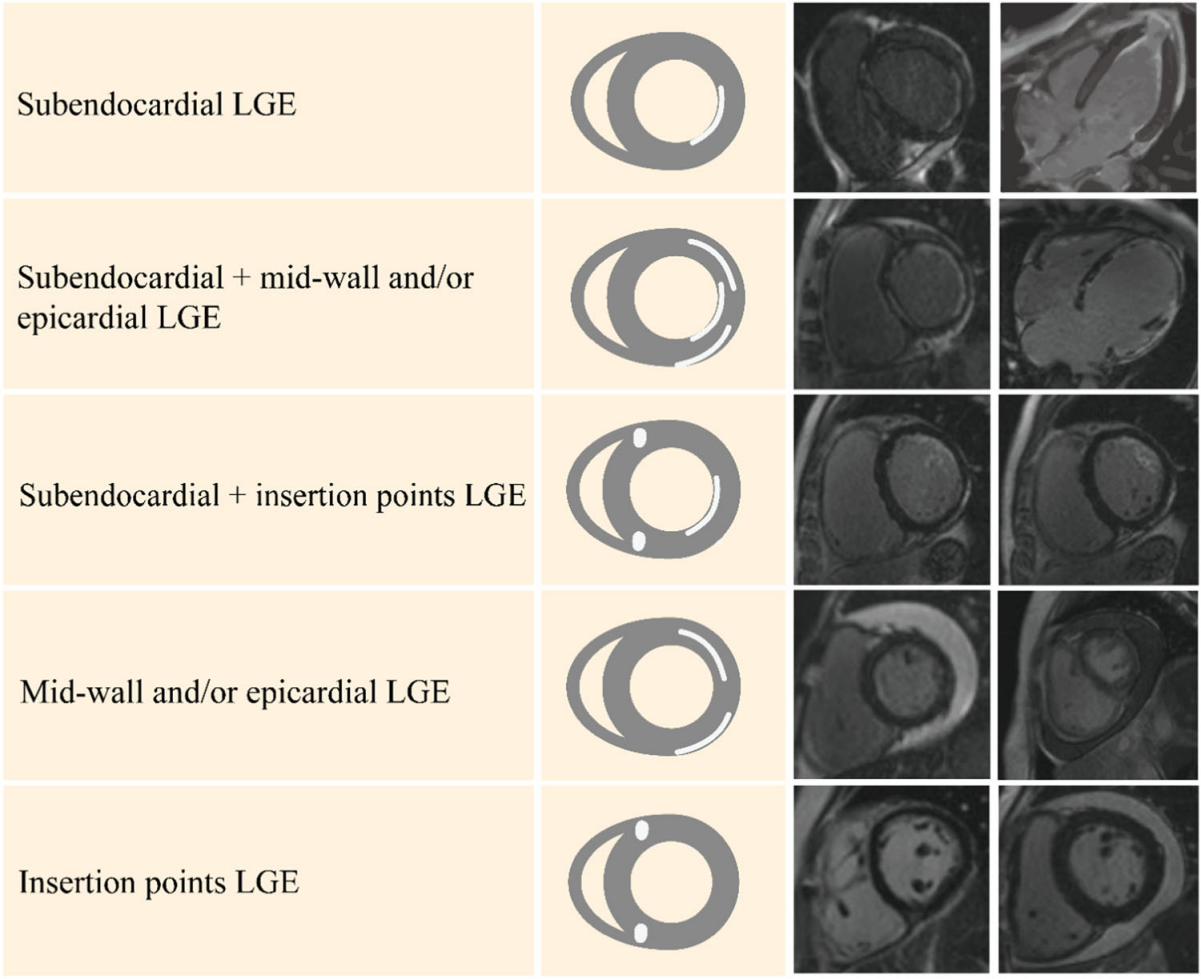
Examples of late gadolinium enhancement patterns in all patients. LGE, late gadolinium enhancement.

The exclusion criteria were as follows: (1) significant coronary arterial stenosis (≥ 50%) on invasive coronary angiography (CAG) or coronary computed tomography angiography (CCTA) (*n* = 19); (2) recognized myocardial infarction (MI), defined as a history of MI corroborated by evidence in medical documentation or the presence of coronary distributive subendocardial LGE (*n* = 8); (3) concomitant congenital heart disease, amyloidosis, viral myocarditis, hypertrophic cardiomyopathy, constrictive pericarditis, and congenital myopathy (*n* = 10); and (4) cancer history (*n* = 1). The workflow is shown in Supporting Information S1: Figure 1. This study was approved by the ethics committee of Peking Union Medical College Hospital (approval number: JS‐2452) and registered at clinicaltrial.gov (NCT03885375). Informed consent was waived due to the retrospective nature of the study.

### 2.2 CMR and Echocardiographic Imaging

Image acquisition and analysis are detailed in the Supplementary Methods. The presence, pattern, and location of LGE were independently assessed by two experienced cardiologists who were blinded to the clinical and echocardiographic data. Discrepancies were resolved by consensus. LGE was considered present if it was observed in both the long‐ and short‐axis planes. The extensive pattern was defined as LGE involving ≥ 3 segments continuously. The LV LGEse pattern was defined as LGE involving the LV subendocardium that did not correspond to a coronary vascular distribution in patients without evidence of MI, which could coexist with LGE involving other layers of the myocardium and other chambers of the subendocardium.

### 2.3 Follow‐Up

The follow‐up duration was defined from the initial CMR evaluation to the most recent evaluation by a clinic visit or telephone review or to the most recent event. Patients were followed for a median of 776 days (interquartile range [IQR], 395–1405 days), and 22 were lost to follow‐up. Endpoint events were a composite of cardiac death, heart failure‐related admission, cardiogenic shock, and appropriate pacemaker or implantable cardioverter‐defibrillator therapy.

### 2.4 Statistical Analysis

The data are presented as the mean ± standard deviation, median (IQR), or n (%) as appropriate. Comparisons between two groups were made using Student's *t* test for normally distributed continuous variables or via the Mann‒Whitney *U* test for nonnormally distributed variables. Frequencies were compared with the chi‐square test or Fisher's exact test, as appropriate. The association between the LV LGEse subtype and adverse cardiac outcomes was evaluated using Cox proportional hazard regression models. Univariable analysis was initially performed among LV LGEse and potential confounders, followed by bidirectional stepwise multiple Cox regression analysis starting with all of the significant factors from the univariable analysis. Then, multivariable Cox regression was performed with the above screened variables.

Subgroup analyses were conducted to further explore the impact of ARD types on outcomes. To exclude the impact of cardiac wall motion abnormalities and LV ejection fraction (LVEF) on outcomes, subgroup analyses of patients with LVEF > 50% and cardiac wall motion abnormalities were also performed. Another sensitivity analysis was performed by excluding patients with LGEse but without CAG or CCTA. In patients without LV subendocardial LGE, other cardiac level‐involved LGE (e.g., myocardium, epicardium, and insertion points of the right ventricle) (Figure 1) subgroup analyses were performed. Two‐tailed *p* < 0.05 was assumed to indicate statistical significance. Statistical analyses were performed using the R packages (version 4.1.2).

## 3 Results

### 3.1 Overall Cohort Characteristics

**Figure 2:**
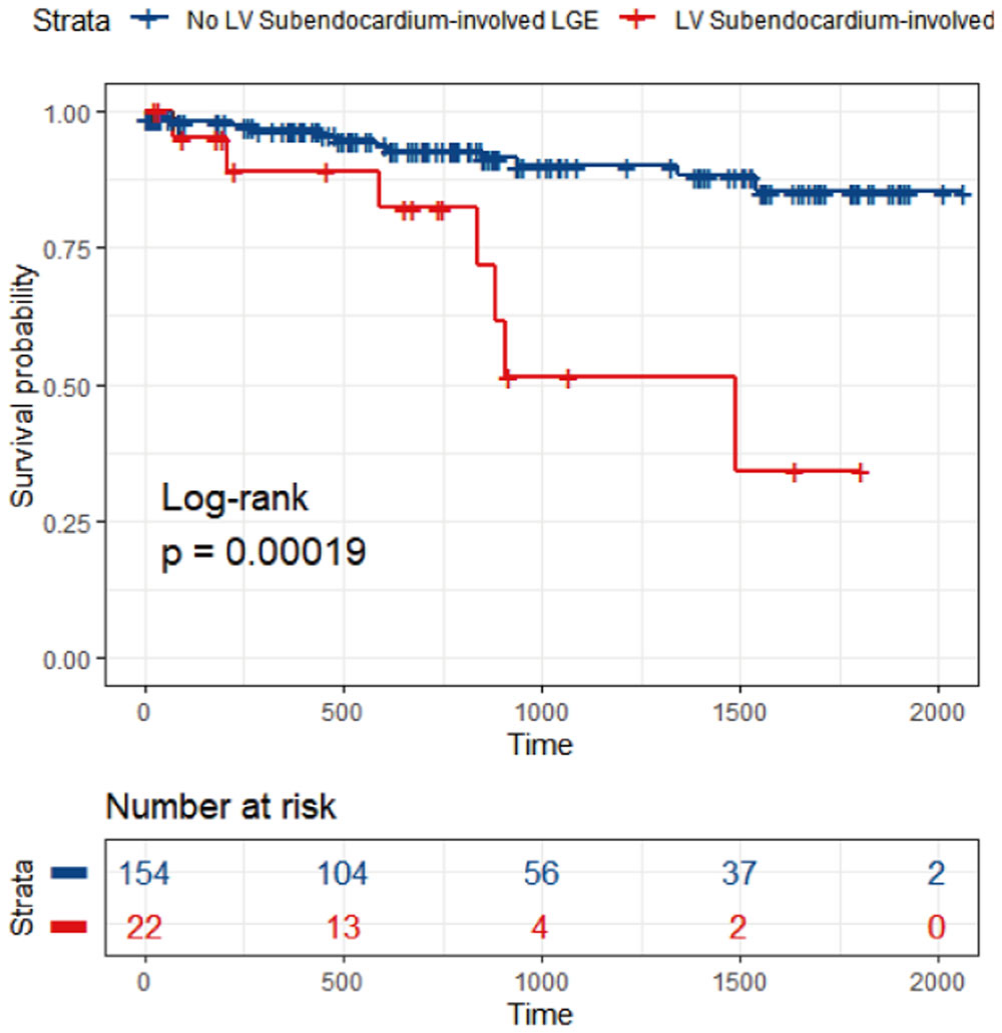
Kaplan‒Meier curves for composite events in all patients. Curves for composite event‐free survival are stratified by LV subendocardium‐involved LGE for all patients. LV, left ventricle; LGE, late gadolinium enhancement.

A total of 176 ARD patients were ultimately enrolled (age: 39 years [IQR, 29–50 years]; 140 women). The patients' baseline characteristics are outlined in Tables 1 and 2. The most prevalent ARDs in this cohort were SLE (*n* = 66), idiopathic inflammatory myopathies (*n* = 51), and systemic vasculitis (*n* = 33). Other ARDs include systemic sclerosis (*n* = 10), Sjogren's syndrome (*n* = 5), rheumatoid arthritis (*n* = 3), antiphospholipid syndrome (*n* = 2), relapsing polychondritis (*n* = 2), mixed connective tissue disease (*n* = 2), systemic sarcoidosis (*n* = 1) and adult‐onset Still's disease (*n* = 1). The distribution and characteristics of ARDs in the study are shown in Supporting Information S1: Figure 2 and Supporting Information S1: Table 1.

**Table 1:** Baseline characteristics of the study patients.

	All Patients (*n* = 176)	LV Subendocardial Involvement (*n* = 22)	No LV Subendocardial Involvement (*n* = 154)	*P* value
**Baseline data**				
Age, y	39 (29–50)	41 (29–49)	39 (29–51)	0.833
Female	140 (80)	8 (36)	132 (86)	**< 0.001**
Disease duration, m	16 (4–72)	24 (12–46)	15 (4–73)	0.370
ARD types				**0.001**
SLE	66 (38)	2 (9)	64 (42)	
IIMs	51 (29)	5 (23)	46 (30)	
SV	33 (19)	9 (41)	24 (16)	
Other	26 (15)	6 (27)	20 (13)	
NYHA functional class III/IV	33 (19)	6 (27)	27 (18)	0.258
Smoking	21 (12)	5 (23)	16 (10)	0.149
**Symptoms**				
Chest pain	18 (10)	3 (14)	15 (10)	0.476
Chest distress	77 (44)	14 (64)	63 (41)	0.075
Palpitation	34 (19)	6 (27)	28 (18)	0.384
Tachypnea	61 (35)	12 (55)	49 (32)	0.063
**Laboratory test findings**				
hsCRP, mg/L	3 (1–9)	3 (1–6)	3 (1–10)	0.766
ESR, mm/h	17 (6–34)	12 (3–16)	20 (7–35)	**0.024**
cTnI, ng/mL	0.04 (0.02–0.17)	0.10 (0.02–0.42)	0.03 (0.02–0.16)	0.110
NT‐proBNP, pg/mL	629 (144–2494)	1448 (681–2901)	575 (131–2238)	0.064
**Comorbidities**				
Diabetes	7 (4)	1 (5)	6 (4)	1.000
Hypertension	32 (18)	2 (9)	30 (20)	0.375
**Medications**				
ACEI/ARB	53 (30)	10 (46)	43 (28)	0.153
Beta‐blocker	79 (45)	11 (50)	68 (44)	0.775
Diuretic agent	66 (38)	15 (68)	51 (33)	**0.003**
Statin	30 (17)	5 (23)	25 (16)	0.543

Abbreviations: ACEI/ARB, angiotensin‐converting enzyme inhibitor/angiotensin receptor blocker; ARD, autoimmune rheumatic disease; cTnI, troponin I peak; NT‐proBNP, N‐terminal pro‐brain natriuretic peptide; ESR, erythrocyte sedimentation rate; hsCRP, hypersensitive C‐reactive protein; IIMs, idiopathic inflammatory myopathies; LV, left ventricular; NYHA, New York Heart Association; SLE, systemic lupus erythematosus; SV, systemic vasculitis.

**Table 2:** CMR and echocardiographic characteristics of the study patients.

	All Patients (*n* = 176)	LV Subendocardial Involvement (*n* = 22)	No LV Subendocardial Involvement (*n* = 154)	*P* value
**CMR**				
**Structural and functional parameters**				
LV end‐diastolic volume index, mL/m^2^	75 (63–92)	78 (70–126)	75 (61–88)	**0.043**
LVEF, %	59 (51–66)	50 (33–60)	60 (53–67)	**0.001**
T2W	13 (7)	4 (18)	9 (6)	0.062
Cardiac wall motion abnormality	82 (47)	17 (77)	65 (42)	**0.004**
**LGE pattern**				
Other subendocardial LGE	7 (4)	5 (23)	2 (1)	**< 0.001**
Extensive LV endocardial LGE	3 (2)	3 (14)	0 (0)	**0.002**
Other extensive LGE	3 (2)	2 (9)	1 (1)	**0.042**
**Echocardiography**				
**LV function**				
E/e'	9 (7–11)	10 (7–13)	9 (7–11)	0.531
GLSendo, %	−23 (‐27 to ‐18)	−21 (−24 to −16)	−23 (−27 to −19)	0.195
GCS, %	−23 ± 7	−19 ± 8	−23 ± 7	**0.016**
GWE, %	90 (84–93)	88 (79–93)	90 (85–93)	0.185
**Other chamber function**				
RV global strain, %	−18 ± 6	−17 ± 8	−19 ± 6	0.245
LA SCT, %	−11 (−7 to −15)	−9 (−6 to −14)	−12 (−8 to −15)	0.355

Abbreviations: CMR, cardiac magnetic resonance; GCS, global circumferential strain; GLSendo, global longitudinal strain of the subendocardium; GWE, global work efficiency; LA, left atrial; LGE, late gadolinium enhancement; LV, left ventricular; LVEF, left ventricle ejection fraction; RV, right ventricle; SCT, contraction strain; T2W, T2 weighted imaging.

LGE was found in 109 patients (62%), 24 of whom had subendocardium‐involved LGE. Their medical documentation was thoroughly double‐checked, and contaminant MI was excluded on the basis of symptoms (chest pain, which travels from the left arm to the neck, shortness of breath, sweating, nausea), cardiac enzymes (a rise and/or fall in cardiac troponin values with at least one value above the 99th percentile upper reference limit), electrocardiography (development of pathological Q waves, new ischemic ECG changes), and imaging changes (new regional wall motion abnormalities in a pattern consistent with an ischemic etiology).

### 3.2 Demographic and Clinical Characteristics

The baseline characteristics of the patients are presented in Table 1. According to the presence and location of LGE, the cohort was dichotomized into LV LGEse (*n* = 22) and no LV LGEse (*n* = 154) groups. Compared with the group without LV LGEse, the group with LV LGEse had a greater proportion of men (64% vs. 14%; *p* < 0.001) and a higher rate of diuretic use (68% vs. 33%; *p* = 0.003). The main symptom in both groups was chest distress (64% vs. 41%; *p* = 0.075), followed by tachypnea (55% vs. 32%; *p* = 0.063). We found no evidence of a difference in terms of cardiac troponin I or N‐terminal pro‐brain natriuretic peptide levels between patients with and without LV LGEse (both were observed *p* > 0.05).

### 3.3 CMR and Echocardiographic Findings

As presented in Table 2, the LV LGEse group had a lower LVEF (50% [IQR, 33%–60%] vs. 60% [IQR, 53%–67%]; *p* = 0.001), larger LV end‐diastolic volume index (78 [IQR, 70–126] vs. 75 [IQR, 61–88]; *p* = 0.043), and higher rate of cardiac wall motion abnormalities (77% vs. 42%; *p* = 0.004). The main echocardiographic finding was a lower level of global circumferential strain (GCS) (−19 ± 8 vs. −23 ± 7; *p* = 0.016) in the LV LGEse cohort than in the non‐LV LGEse cohort. No significant differences were observed in terms of E/e' or left atrium contraction strain among these groups (*p* > 0.05).

LV LGEse was observed in 13% of all autoimmune disease patients with clinically suspected cardiac involvement (22/176) and in 20% of patients with LGE (22/109). Other chamber LGEse was observed in seven patients, five of whom had coexisting LV LGEse. Among the patients with LV LGEse, 14% exhibited extensive LV LGEse (3/22). Other extensive LGEs were observed in three patients, two of whom had coexisting extensive LV LGEse. The regions most severely affected by left ventricular global longitudinal strain of the subendocardium (GLSendo) in autoimmune disease patients were the basal segments, particularly the septal, anterior, and posterior segments, which appeared to form a trefoil shape. In the LV LGEse group, the basal posterior, medial posterior, and basal anterior segments were more vulnerable than in the groups without LV subendocardium involvement, which made the trefoil sign clearer (Supporting Information S1: Figure 3).

### 3.4 Associations Between LGE and Prognosis

Over a median follow‐up of 776 days (IQR, 395–1405 days), 20 patients experienced composite events (Supporting Information S1: Table 2). Compared with the no LV LGEse group, the LV LGEse group had a greater incidence of the composite endpoint (Figure 2). Univariable Cox regression analysis confirmed that LV LGEse was a significant univariable predictor of the composite endpoint (hazard ratio, 4.91; CI, 1.94–12.37; *p* < 0.001). Starting with the significant factors from the univariable models (LV LGEse, LVEF, cardiac wall motion abnormality, and GCS), the bidirectional stepwise multiple Cox regression analysis screened out two variables, namely LV LGEse and cardiac wall motion abnormalities. Then, multivariable Cox regression was performed with the screened variables. After adjustments for cardiac wall motion abnormalities, the risk of LV LGEse remained significant (Table 3).

**Table 3:** Univariable and multivariable Cox model analyses assessing the relationships between clinical and imaging data and outcomes.

	Univariable		Multivariable	
	Hazard ratio	*P* value	Hazard ratio	*P* value
LV Subendocardial Involvement	4.91 (1.94–12.37)	**< 0.001**	3.47 (1.34–8.98)	**0.010**
Sex	0.96 (0.32–2.88)	0.945		
ESR	0.99 (0.97–1.01)	0.421		
ARD types	1.05 (0.84–1.30)	0.675		
LV end‐diastolic volume index	1.01 (1.00–1.02)	0.060		
LVEF	0.96 (0.94–0.99)	**0.004**		
Cardiac wall motion abnormality	4.45 (1.48–13.31)	**0.008**	3.47 (1.12–10.71)	**0.031**
GCS	0.94 (0.88–1.00)	**0.047**		

Abbreviations: ARD, autoimmune rheumatic disease; GCS, global circumferential strain; ESR, erythrocyte sedimentation rate; LV, left ventricular; LVEF, left ventricle ejection fraction.

In patients with ARD, the positive association between the presence of LV LGEse and composite events was found to be predominant in patients with SLE, IIM, and other ARDs (Supporting Information S1: Figure 4). Subgroup analyses of patients with LVEF > 50% and cardiac wall motion abnormalities showed that patients with LV LGEse still had a greater risk of composite events. To further verify the association between LV LGEse and adverse outcomes, a sensitivity analysis was performed, excluding patients with LGEse but without CAG or CCTA; LV LGEse still had significant prognostic value (Supporting Information S1: Table 3) (Supporting Information S1: Figure 5).

Patients with autoimmune disease may also present with LGE involving other cardiac layers. In our cohort, LGE involving other cardiac layers was mostly located in the left ventricle, including the LV myocardium (*n* = 56), LV epicardium (*n* = 1), LV subepicardium (*n* = 10), and RV insertion points (*n* = 28). However, we found no significant difference between LGE involving these cardiac layers and adverse outcomes in the subgroup analyses without 22 LV subendocardial LGE patients (Supporting Information S1: Table 4) (Supporting Information S1: Figure 6).

## 4 Discussion

In this study, we retrospectively enrolled ARD patients with clinically suspected cardiac involvement and explored and compared their clinical characteristics, imaging features, and outcomes. The main findings were as follows: (1) The ARD group with LV LGEse had a greater proportion of men and a higher rate of diuretic use. (2) In the ARD group, patients with LV LGEse had a lower LVEF, greater left ventricular end‐diastolic volume index, and higher rate of cardiac wall motion abnormalities. (3) LV LGEse has an impact on prognosis, with a four‐fold increased risk of adverse outcomes.

ARDs are heterogeneous disorders in which tolerance to self‐antigens is compromised, leading to inappropriate immune reactivity and chronic inflammation. Although novel therapeutic strategies for ARDs have led to significant reductions in disease‐associated mortality, patients with ARDs still have a lower average life expectancy than does the general population [13]. This difference can be partially explained by the increased incidence of cardiovascular disease in the population, especially the early age of onset and accelerated course of atherosclerotic disease, which has variable but clearly demonstrated adverse effects on prognosis [14].

More recently, CMR‐derived LGE, which allows for detailed evaluation of cardiac tissue characterization, has been shown to be valuable for the early diagnosis of cardiovascular abnormalities in patients with ARD [15, 16]. S Mavrogeni et al. discovered that, in an SLE cohort of 20 patients, abnormalities on CMR imaging were observed in 80% of the patients, compared to 15% with abnormal ECG [16]. In an EGPA cohort of 176 patients, Silvia Sartorelli et al. reported that abnormalities on CMR imaging were observed in 80% of the patients, compared to 49% with abnormal ECG results and 53% with abnormal echocardiography results [10]. The presence of LGE was significantly associated with the occurrence of major adverse cardiovascular events after a follow‐up period of 61 months [10]. Nevertheless, another recent study reported no significant association between LGE and adverse outcomes in 102 ARD patients during a follow‐up of 25 months (*p* = 0.051), possibly due to the relatively small sample size and short follow‐up time [17].

In most ARD patients, the predominant LGE pattern is myocardial and epicardial involvement [4, 5, 6, 7, 8, 9]. Edward Hulten et al. identified seven cohort studies of 694 patients with known sarcoidosis with suspected cardiac involvement, indicating that the typical LGE pattern in cardiac sarcoidosis patients is midmyocardial and subepicardial involvement [7]. Atsuma Nishiwaki and colleagues studied 52 primary Sjögren syndrome patients [5] and 51 idiopathic inflammatory myopathy patients [4]. They discovered that the most common locations of LGE in the primary Sjögren syndrome group were the myocardium and epicardium [5], whereas in the idiopathic inflammatory myopathy group, it was the myocardium [4]. Nevertheless, other types of LGE may occur and have roles in ARD patients that have long been overlooked. LV LGEse is an underrecognized feature in ARD patients and accounts for 13% of our cohort of 176 ARD patients.

The exact mechanisms of LV LGEse in ARD patients remain unclear. There are several hypotheses. First, inflammation‐induced premature and accelerated atherosclerosis leads to atherosclerotic vascular plaque formation in coronary arteries and a potentially early onset of coronary heart disease [18]. In rheumatoid arthritis, the release of pro‐inflammatory cytokines, such as tumor necrosis factor‐α, leads to endothelial dysfunction and to increased expression of chemoattractants and adhesion molecules [19], which promote leukocyte infiltration, proliferation and activation in the subendothelial space [20]. Similarly, increased levels of CRP and pro‐inflammatory cytokines have been found to be associated with the development of premature atherosclerosis in patients with SLE [21, 22]. In MI, the presence of subendocardial LGE can be attributed to certain pathophysiological mechanisms, such as coronary artery stenosis, embolism, and coronary spasm [23]. It commonly manifests as stripe‐like LGE involving the subendocardium along the coronary artery distribution [24]. However, LGE in ARD patients does not present the typical pattern [25]. Therefore, the possibility of coronary artery disease in ARD patients should be carefully assessed. Second, inflammation‐related microvascular dysfunction, expressed as either an inability of the microcirculation to dilate appropriately in response to increased myocardial oxygen demand or as coronary microvascular spasm, leads to recurrent ischemia and fibrosis over time [18]. A meta‐analysis of 709 ARD patients with a low probability of CAD revealed a significantly lower coronary flow reserve (CFR) in these patients than in 650 controls. Patients with prevalent autoimmune features such as SLE have a significantly lower CFR than patients with mixed autoimmune and autoinflammatory features such as rheumatoid arthritis [26]. Another study reported that foci of myocardial fibrosis were found in 44% of systemic sclerosis autopsied patients without coronary stenosis, which is frequent in the subendocardium. These lesions may develop from transient nonperfusion with reperfusion [27]. Third, the particularity of the LV structure and mechanics are proposed to explain myocardial ischemia in ARD patients. The left ventricle is the main pumping chamber of the heart and pumps blood at higher pressure, as it faces a much greater workload and mechanical afterload [28]. The left ventricle is subjected to extreme pressure fluctuations and compression during both systole and diastole, making the left ventricle vulnerable to early structural changes, including fibrosis. Simon Greulich et al. reported that, in an antineutrophil cytoplasmic antibody‐associated vasculitides cohort of 34 patients, a lower LVEF was observed in LGE‐positive patients than in LGE‐negative patients (59% vs. 69%, *p* = 0.01) [29]. In a sarcoidosis cohort of 21 patients, the same research team reported that LGE‐positive patients had a significantly lower LVEF (49% vs. 66%, *p* = 0.04) and a larger LV end‐systolic volume (85 mL vs. 49 mL, *p* = 0.02) than LGE‐negative patients did [30]. Fourth, a longer duration of microvascular abnormalities could escalate the progression of decompensation. Tatiana S. Rodriguez‐Reyna et al. noted that in systemic sclerosis patients with a disease duration > 5 years, there was a trend toward an association between the presence of myocardial fibrosis and perfusion abnormalities (*p* = 0.07). This association was unlikely to be age related since it was not associated with either factor [31].

Given the relative paucity of LGE data in Asian ARD populations, the present study provides an overview of the prevalence of LV LGEse in a consecutive Asian cohort. To our knowledge, this is the first study to demonstrate associations between poor prognosis and specific LGE feature in ARD patients. The prognostic significance remained after adjusting for LVEF. On the one hand, myocardial necrosis or fibrosis generated by microvascular dysfunction, which is one of the causes of LV subendocardial involvement, has been shown to be an important cause of early heart failure [32]. On the other hand, because subendocardial contraction is greatest in the longitudinal plane from the mitral annulus to the apex, the impairment of subendocardial muscle function caused by fibrosis can affect long‐axis dysfunction in the left ventricle [33]. Considering the ischemic susceptibility of the LV chamber and subendocardium, the LV subendocardial involvement observed in ARD patients might be an early marker reflecting severe cardiac conditions and heart failure.

LV subendocardial contraction is greatest in the longitudinal plane and can be measured using GLSendo technology, which employs two‐dimensional speckle tracking. The segment GLSendo model of ARD patients displayed a trefoil shape characterized by basal segment impairment, particularly in the septum and anterior and posterior regions. The GLSendo trefoil shape became even more pronounced in the LV LGEse group. Heterogeneity in the structure and function of the coronary microcirculation could account for these findings: blood flow and contractile function tend to be greater at the apex and anterior LV wall than at the base and posterior wall, respectively [34]. LV subendocardial involvement, resulting from microvascular dysfunction and/or microvascular dysfunction‐induced myocardial necrosis or fibrosis, may exacerbate the already existing heterogeneity in the coronary microcirculation. With respect to LGE involving other cardiac layers (e.g., the myocardium and RV insertion points), according to the current evidence, LGE does not indicate a worse prognosis, likely owing to the high prevalence of most ARDs [4, 5, 6, 7, 8, 9] and the relatively favorable outcomes of ARDs. Apart from LGE, newer CMR protocols, including T1 and T2 mapping, may also add value for the assessment of myocardial tissue characteristics in ARD patients [29, 35, 36]. Nevertheless, data on patient prognosis are scarce, and additional research is needed.

### 4.1 Study Limitations

First, the number of patients enrolled in this single‐center cohort study was small, so the validation of our findings in multicenter cohorts is warranted. However, in our cohort, all patients were assessed by CMR and echocardiography, which are relatively comprehensive noninvasive assessment data, especially in the Asian population. Second, our current research includes various types of ARDs. Therefore, further studies evaluating each ARD separately are needed to better clarify the role of LV LGEse involvement in predicting outcomes for each individual disease. However, in the SLE, IIM, and other ARD subgroup analyses, LGE involving the LV subendocardium was still significantly associated with adverse outcomes (*p* < 0.05). Third, this retrospective study did not evaluate microvascular perfusion, which can be assessed by positron emission tomography, computed tomography, or CMR stress testing. Given the finding in our cohort of an association between poor prognosis and LV LGEse features in ARD patients, the potential correlation between microvascular dysfunction and the LV LGEse pattern should be explored. Fourth, advanced imaging techniques (myocardial T1, T2, and extracellular volume mapping) were not included because of the lack of consistent and standardized mapping data across the entire study period. These mapping methods offer a more comprehensive assessment of myocardial tissue characteristics in ARD patients and could provide additional prognostic information. Future studies incorporating these advanced imaging techniques are warranted. Fifth, we included 10 patients with LGEse who did not undergo CAG/CCTA to account for the real‐world prevalence of LGE features in ARD populations and to reduce age‐ and symptom severity‐related selection bias. After excluding patients with LGEse but without CAG or CCTA, LGE involving the LV subendocardium still exhibited a significant association with adverse outcomes, with a greater hazard ratio (3.27 vs. 3.20) after adjustment for the LVEF. Sixth, while the number of outcome events was small, we conducted a bidirectional stepwise multiple Cox regression analysis among the significant factors from the univariable models, which screened out two variables. After adjusting the another, the LV LGEse still has a significant prognostic effect.

## 5 Conclusions

In ARD patients, CMR‐detected LV subendocardial LGE was more strongly associated with a higher proportion of men, lower LVEF, higher rates of cardiac wall motion abnormalities, and more adverse outcomes.

## Author Contributions

Wei Chen and Yining Wang were involved in the study conception and design; Danni Wu, Xiao Li, Tianchen Guo, Xiaojin Feng, and Xinhao Li collected the data and performed the data entry. Danni Wu analyzed and interpreted the data. Danni Wu prepared the initial draft of the manuscript. Danni Wu and Wei Chen prepared the final drafts of the article. All of the authors critically reviewed and approved the final draft and are responsible for the content and similarity index of the manuscript. All of the authors agree to be accountable for all aspects of the work.

## Conflicts of Interest

The authors declare no conflicts of interest.

## Supporting information

Supporting information.

## Data Availability

The data supporting this study's findings are available on request from the corresponding author [Yining Wang]. The data are not publicly available because they contain information that could compromise the privacy of research participants.
