# Supplementary material for: Unmasking the Hidden Threat: The Role of Left Ventricular Subendocardial Involvement in Autoimmune Rheumatic Disease
Source: Clin Cardiol. 2025 Jan 1;48(1):e70069. doi: 10.1002/clc.70069 (PMC11693844; doi:10.1002/clc.70069)
Supplement: Supplementary file 1 — Supporting information. [file CLC-48-e70069-s001.docx]

**SUPPLEMENTAL METHODS**

**CLASSIFICATION CRITERIA OF ARDs**

Autoimmune diseases, including systemic lupus erythematosus (American College of Rheumatology (ACR) 1997 criteria^1^), idiopathic inflammatory myopathies (Bohan and Peter criteria^2^), systemic vasculitis (ACR 1990 criteria^3^), systemic sclerosis (ACR 1980 criteria^4^), antiphospholipid syndrome (updata 2006 criteria^5^), Sjogren’s syndrome (American-European Consensus Group (AECG) 2002 criteria^6^), rheumatoid arthritis (ACR 1987 criteria^7^), relapsing polychondritis (clinical criteria^8^), mixed connective tissue disease (clinical criteria^9^), ankylosing spondylitis (New York 1984 criteria^10^), immunoglobulin-G4-related disease (comprehensive diagnostic 2012 criteria^11^), systemic sarcoidosis (American Thoracic Society (ATS) 2020 criteria^12^), and adult-onset Still’s disease (clinical criteria^13^) were diagnosed according to the indicated classiﬁcation criteria.

**CMR IMAGE**

CMR imaging was performed with a 3.0-T MR scanner (MAGNETOM Skyra, Siemens Healthineers) using electrocardiographic and respiratory gating. The data were read and analyzed using a postprocessing workstation (IntelliSpace Portal; Philips Medical Systems).

Using segmented balanced steady-state free precession sequences, cines of three long-axis slices and a stack of 9–12 short-axis slices with full coverage of the left ventricle were acquired to evaluate volumes and functional parameters. At the same position as the cines in end-diastole, LGE images were obtained 10–15 min after intravenous administration of gadolinium-based contrast medium (gadobenate dimeglumine, Beijing BEILU Pharmaceutical Co., Ltd.) at a dose of 0.15 mmol/kg.

The typical imaging parameters for segmented balanced steady-state free precession sequences were repetition time (TR) = 3.3 ms, echo time (TE) = 1.43 ms, flip angle = 55⁰–70⁰, voxel size = 1.6 × 1.6 × 6.0 mm^3^, temporal resolution = 45.6 ms, field of view = 320 × 320 mm^2^, and matrix = 164 × 157. The typical imaging parameters for two-dimensional phase-sensitive inversion-recovery gradient‒echo pulse sequences were a TR = 5.2 ms, a TE = 1.96 ms, a flip angle = 20°, and a voxel size = 1.4 × 1.4 × 8.0 mm^3^.

**ECHOCARDIOGRAPHIC IMAGE**

Echocardiography was performed with a General Electric Vivid 9 (GE Medical System, Milwaukee, Wisconsin) with a 3.5 MHz transducer in accordance with the recommendations of the European Association of Echocardiography/American Society of Echocardiography^14^. Echocardiographic data were read and analyzed using General Electric EchoPAC software (version 113).

Three consecutive heart cycles were recorded. Pulsed-wave Doppler imaging was performed in the apical four-chamber view with the sample volume placed between the mitral leaflet tips to obtain the diastolic mitral early velocity (E). Pulsed-wave early diastolic tissue Doppler velocity (e’) values were determined from the apical four-chamber view at the lateral and septal regions of the mitral annulus. Left ventricular global longitudinal strain of the subendocardium (GLSendo) and myocardial work were measured via two-dimensional speckle tracking. The average of the three apical views was calculated. The methods, including inter- and intraobserver variability, have been described in detail elsewhere^15^, and the results showed good agreement. The intraobserver and interobserver intraclass correlation coefficients were 0.95 and 0.86, respectively. Right ventricular global strain and left atrial systolic strain were also measured. Global circumferential strain (GCS) data were obtained from short-axis views.

**SUPPLEMENTAL REFERENCES**

1. Hochberg MC. Updating the American College of Rheumatology revised criteria for the classification of systemic lupus erythematosus. *Arthritis Rheum.* 1997;40(9):1725.

2. Bohan A, Peter JB. Polymyositis and dermatomyositis (second of two parts). *N Engl J Med.* 1975;292(8):403-407.

3. Hunder GG, Arend WP, Bloch DA, et al. The American College of Rheumatology 1990 criteria for the classification of vasculitis. Introduction. *Arthritis Rheum.* 1990;33(8):1065-1067.

4. Preliminary criteria for the classification of systemic sclerosis (scleroderma). Subcommittee for scleroderma criteria of the American Rheumatism Association Diagnostic and Therapeutic Criteria Committee. *Arthritis Rheum.* 1980;23(5):581-590.

5. Miyakis S, Lockshin MD, Atsumi T, et al. International consensus statement on an update of the classification criteria for definite antiphospholipid syndrome (APS). *J Thromb Haemost.* 2006;4(2):295-306.

6. Vitali C, Bombardieri S, Jonsson R, et al. Classification criteria for Sjögren's syndrome: a revised version of the European criteria proposed by the American-European Consensus Group. *Ann Rheum Dis.* 2002;61(6):554-558.

7. Arnett FC, Edworthy SM, Bloch DA, et al. The American Rheumatism Association 1987 revised criteria for the classification of rheumatoid arthritis. *Arthritis Rheum.* 1988;31(3):315-324.

8. Michet CJ, Jr., McKenna CH, Luthra HS, O'Fallon WM. Relapsing polychondritis. Survival and predictive role of early disease manifestations. *Ann Intern Med.* 1986;104(1):74-78.

9. R K, G S. Kasukawa R, Sharp G, editors. Mixed connective tissue disease and antinuclear antibodies. Amsterdam: Elsevier; 1987. p. 41–7. *Amsterdam: Elsevier.* 1987(p. 41–7).

10. van der Linden S, Valkenburg HA, Cats A. Evaluation of diagnostic criteria for ankylosing spondylitis. A proposal for modification of the New York criteria. *Arthritis Rheum.* 1984;27(4):361-368.

11. Umehara H, Okazaki K, Masaki Y, et al. Comprehensive diagnostic criteria for IgG4-related disease (IgG4-RD), 2011. *Mod Rheumatol.* 2012;22(1):21-30.

12. Crouser ED, Maier LA, Wilson KC, et al. Diagnosis and Detection of Sarcoidosis. An Official American Thoracic Society Clinical Practice Guideline. *Am J Respir Crit Care Med.* 2020;201(8):e26-e51.

13. Efthimiou P, Paik PK, Bielory L. Diagnosis and management of adult onset Still's disease. *Ann Rheum Dis.* 2006;65(5):564-572.

14. Lang RM, Bierig M, Devereux RB, et al. Recommendations for chamber quantification: a report from the American Society of Echocardiography's Guidelines and Standards Committee and the Chamber Quantification Writing Group, developed in conjunction with the European Association of Echocardiography, a branch of the European Society of Cardiology. *J Am Soc Echocardiogr.* 2005;18(12):1440-1463.

15. Jensen MT, Sogaard P, Andersen HU, et al. Global longitudinal strain is not impaired in type 1 diabetes patients without albuminuria: the Thousand & 1 study. *JACC Cardiovasc Imaging.* 2015;8(4):400-410.

SUPPLEMENTAL TABLE 1 Characteristics of the Study Patients Based on the Type of ARDs

|  | No. of patients | LV Subendocardial  Involvement | Female | NT-proBNP, pg/mL | LVEF, % | Cardiac wall motion abnormality | GWE, % | Composite events |
| --- | --- | --- | --- | --- | --- | --- | --- | --- |
| SLE | 66 | 2 (3) | 61 (92) | 915 (245-4713) | 58 (53-65) | 35 (53) | 90 (85-93) | 5 (8) |
| IIMs | 51 | 5 (10) | 36 (71) | 317 (91-959) | 58 (15) | 21 (41) | 91 (86-93) | 5 (10) |
| SV | 33 | 9 (27) | 21 (64) | 772 (134-3214) | 61 (53-65) | 13 (40) | 90 (85-94) | 5 (15) |
| **Others** |  |  |  |  |  |  |  |  |
| Systemic  sclerosis | 10 | 5 (50) | 8 (80) | 1900 (620-3505) | 50 (13) | 6 (60) | 86 (8) | 3 (30) |
| Sjogren’s  syndrome | 5 | 0 | 5 (100) | 14860 (16770) | 62 (13) | 3 (60) | 85 (9) | 0 |
| RA | 3 | 1 (33) | 3 (100) | 892 (625) | 67 (2) | 2 (67) | 81 (6) | 1 (33) |
| APS | 2 | 0 | 2 (100) | 556 (81) | 62 (2) | 0 | 89 (0) | 0 |
| RP | 2 | 0 | 1 (50) | 65 (11) | 61 (2) | 0 | 94 (3) | 0 |
| MCTD | 2 | 0 | 2 (100) | 6247 | 46 (25) | 1 (50) | 76 (13) | 0 |
| Systemic  sarcoidosis | 1 | 0 | 1 | 747 | 41 | 1 | 77 | 1 |
| Adult-onset Still’s disease | 1 | 0 | 0 | 312 | 58 | 0 | 96 | 0 |

ARDs = autoimmune rheumatic diseases; SLE = systemic lupus erythematosus; IIMs = idiopathic inflammatory myopathies; SV = systemic vasculitis; RA = rheumatoid arthritis; APS = antiphospholipid syndrome; RP = relapsing polychondritis; MCTD = mixed connective tissue disease

SUPPLEMENTAL TABLE 2 Summary of Adverse Events in the Study Patients

|  | LV Subendocardial  Involvement (n = 22) | No LV Subendocardial  Involvement (n = 154) |
| --- | --- | --- |
| **Composite event** | 7 | 13 |
| Cardiac death | 0 | 2 |
| Heart failure-related admission | 2 | 9 |
| Cardiogenic shock | 3 | 2 |
| Pacemaker or ICD therapy | 2 | 0 |

LV = left ventricular; ICD = implantable cardioverter-defibrillator.

SUPPLEMENTAL TABLE 3 Subanalysis of Patients with LVEF > 50%, Cardiac Wall Motion Abnormalities, and Excluding Patients with Subendocardium-involved LGE but without CAG or CCTA Results

| Subgroup and parameter | LV Subendocardial  Involvement | No LV Subendocardial  Involvement | *P* value |
| --- | --- | --- | --- |
| **LVEF > 50%** |  |  |  |
| No. of patients | 11 | 126 |  |
| Female | 3 (27) | 112 (89) | **<0.001** |
| NT-proBNP, pg/mL | 806 (259-1440) | 430 (122-1510) | 0.501 |
| Cardiac wall motion abnormality | 6 (55) | 37 (29) | 0.099 |
| GWE, % | 93 (90-95) | 91 (86-94) | **0.278** |
| Composite events | 3 (27) | 8 (6) | **0.045** |
| **Cardiac wall motion abnormality** |  |  |  |
| No. of patients | 17 | 65 |  |
| Female | 8 (47) | 54 (83) | **0.004** |
| NT-proBNP, pg/mL | 1519 (611-3215) | 1075 (354-5155) | 0.867 |
| LVEF, % | 42 (30-51) | 51 (43-56) | 0.054 |
| GWE, % | 80 (77-89) | 87 (82-90) | 0.235 |
| Composite events | 7 (41) | 9 (14) | **0.018** |
| **Excluding patients with subendocardium-involved LGE but without CAG or CCTA** |  |  |  |
| No. of patients | 13 | 153 |  |
| Female | 5 (39) | 132 (86) | **<0.001** |
| NT-proBNP, pg/mL | 1448 (611-2901) | 572 (128-2125) | 0.142 |
| LVEF, % | 42 (32-53) | 60 (53-67) | **0.001** |
| Cardiac wall motion abnormality | 11 (85) | 65 (43) | **0.008** |
| GWE, % | 90 (79-93) | 90 (85-93) | 0.570 |
| Composite events | 5 (39) | 13 (9) | **0.006** |

LV = left ventricular; LVEF = left ventricle ejection fraction; LGE = late gadolinium enhancement; CAG = coronary angiography; CCTA = coronary computed tomography angiography; GWE = global work efficiency; NT-proBNP = N-terminal pro-brain natriuretic peptide.

SUPPLEMENTAL TABLE 4 Subanalyses of LGE Involving Other Cardiac Layers after Excluding 22 Patients with LV Subendocardium-involved LGE

| Subgroup and parameter | Involvement | No  Involvement | *P* value |
| --- | --- | --- | --- |
| **LV Myocardium** |  |  |  |
| No. of patients | 47 | 107 |  |
| Female | 41 (87) | 91 (85) | 0.915 |
| NT-proBNP, pg/mL | 748 (181-4749) | 493 (121-1571) | 0.154 |
| LVEF, % | 58 (47-65) | 61 (55-67) | 0.201 |
| Cardiac wall motion abnormality | 26 (55) | 39 (36) | 0.045 |
| GWE, % | 90 (84-92) | 91 (86-94) | 0.083 |
| Composite events | 4 (9) | 9 (8) | 1.000 |
| **LV Subadventitia** |  |  |  |
| No. of patients | 9 | 145 |  |
| Female | 5 (56) | 127 (88) | **0.025** |
| NT-proBNP, pg/mL | 888 (92-4280) | 572 (136-2125) | 0.872 |
| LVEF, % | 50 (44-57) | 61 (54-67) | **0.049** |
| Cardiac wall motion abnormality | 7 (78) | 58 (40) | **0.036** |
| GWE, % | 89 (82-91) | 90 (85-94) | 0.438 |
| Composite events | 0 (0) | 13 (9) | 1.000 |
| **RV insertion part** |  |  |  |
| No. of patients | 24 | 130 |  |
| Female | 20 (83) | 112 (86) | 0.752 |
| NT-proBNP, pg/mL | 1504 (426-5286) | 469 (121-1597) | **0.004** |
| LVEF, % | 57 (52-64) | 61 (53-67) | 0.284 |
| Cardiac wall motion abnormality | 16 (67) | 49 (38) | **0.016** |
| GWE, % | 86 (83-92) | 90 (86-94) | 0.108 |
| Composite events | 4 (17) | 9 (7) | 0.122 |

Abbreviations are the same as those in Table 1 and Table 2.


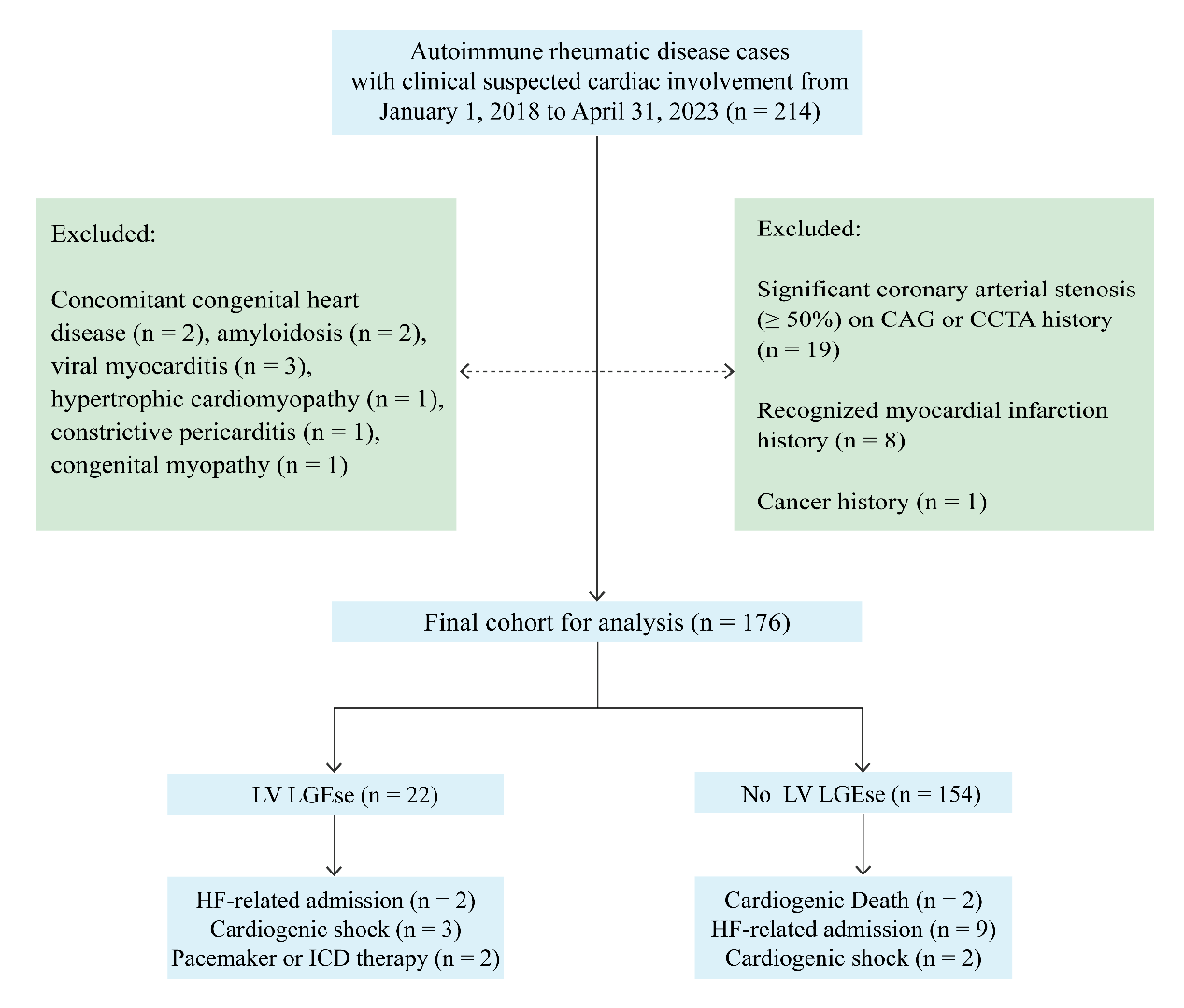


SUPPLEMENTAL FIGURE 1 Study Cohort

Flowchart of patient recruitment. CAG = coronary angiography; CCTA = coronary computed tomography angiography; LV = left ventricular; LGEse = subendocardium-involved late gadolinium enhancement; HF = heart failure; ICD = implantable cardioverter-defibrillator.


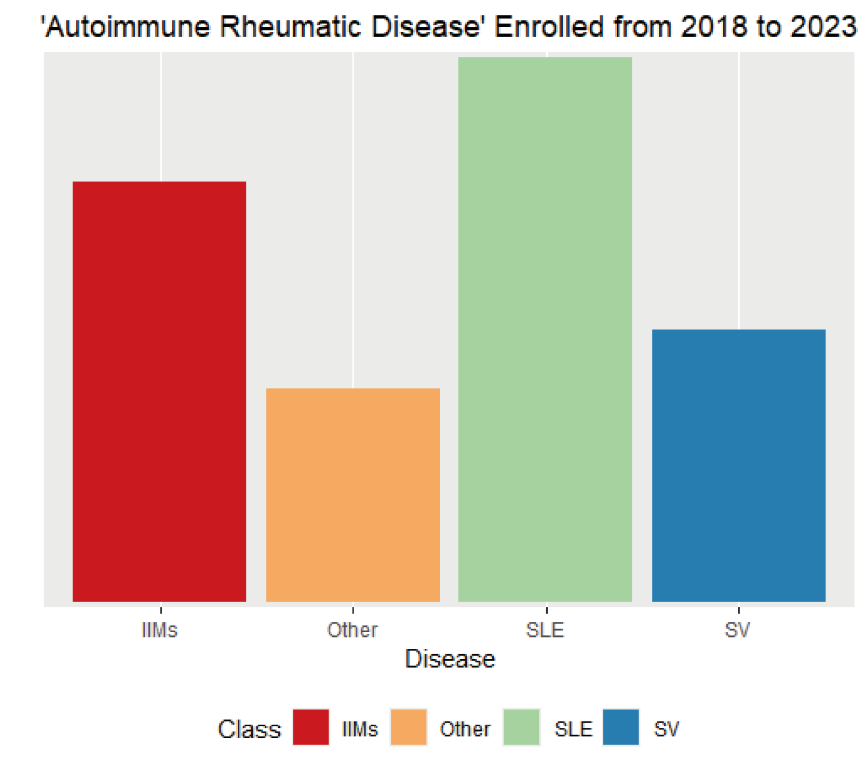


SUPPLEMENTAL FIGURE 2 Distribution of Autoimmune Rheumatic Diseases

SLE = systemic lupus erythematosus; SV = systemic vasculitis; IIMs = idiopathic inflammatory myopathies


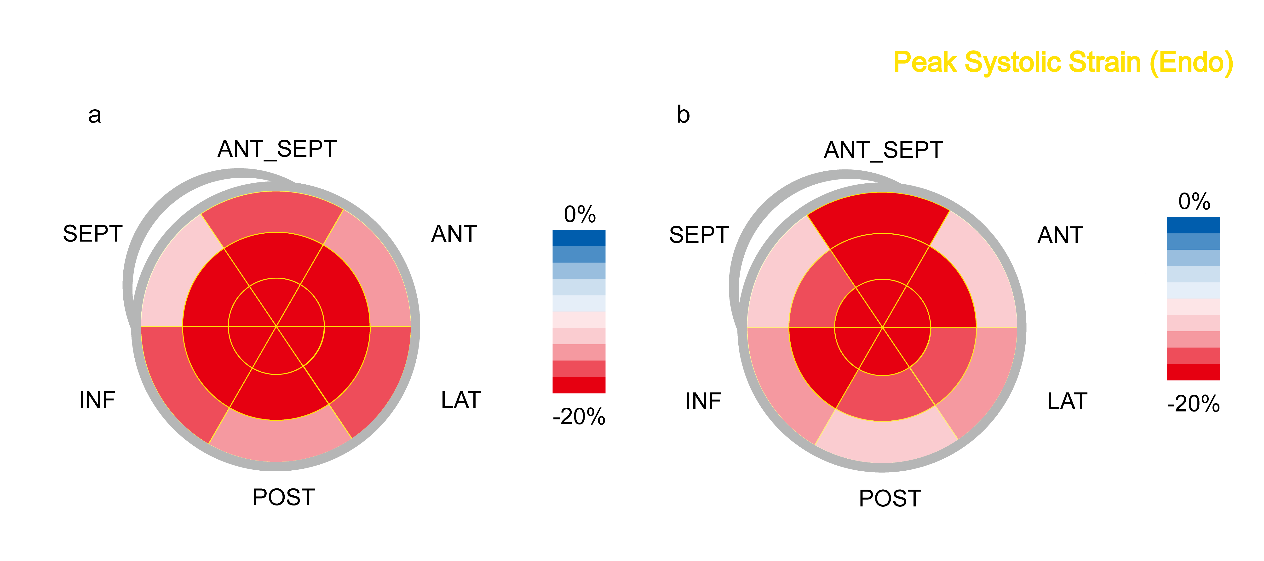


SUPPLEMENTAL FIGURE 3 Distribution of GLSendo

Distribution of subendocardial GLS among all patients in the 18-segment model (**a**) and among LGE patients with LV subendocardial involvement (**b**). Endo = subendocardium GLS; ANT_SEP = anterior septum; ANT = anterior; LAT = lateral; POST = posterior; INF = inferior; SEPT = septum. The other abbreviations are the same as those in Table 2.


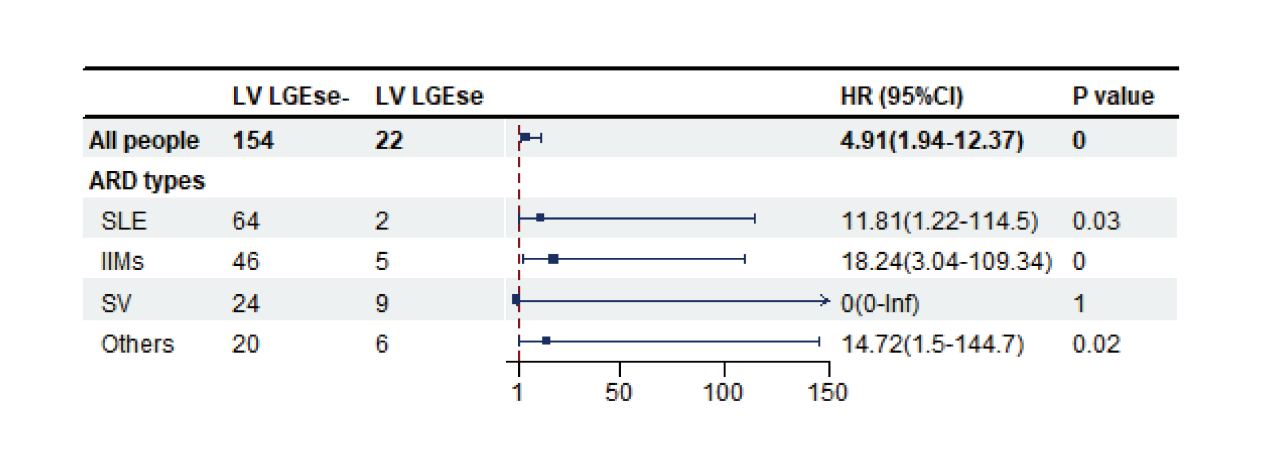


SUPPLEMENTAL FIGURE 4 Subgroup Analyses Based on the Type of Autoimmune Rheumatic Diseases. HRs and 95% CIs were calculated using Cox regression analysis

SLE = systemic lupus erythematosus; SV = systemic vasculitis; IIMs = idiopathic inflammatory myopathies; LV = left ventricular; LGEse = subendocardium-involved late gadolinium enhancement.


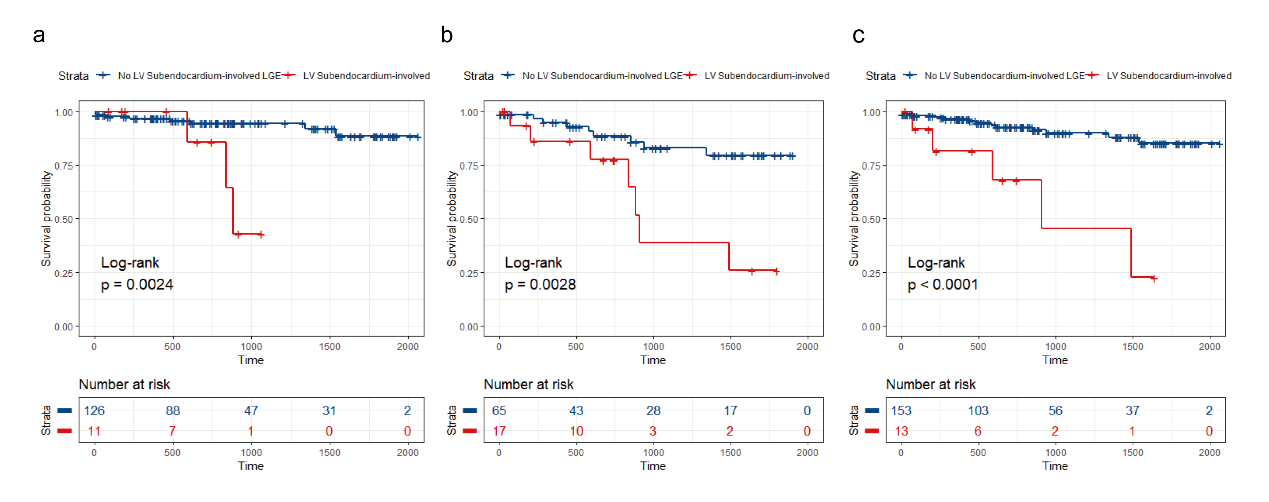


SUPPLEMENTAL FIGURE 5 Kaplan‒Meier Curves for Composite Events in Patients with LVEF > 50%, Cardiac Wall Motion Abnormalities, and excluding Patients with Subendocardium-involved LGE but without CAG or CCTA Results

Curves for composite event-free survival are stratified by LV subendocardium-involved LGE in patients with LVEF > 50% (a), cardiac wall motion abnormalities (b), and excluding patients with subendocardium-involved LGE but without CAG or CCTA results (c). CAG = coronary angiography. CCTA = coronary computed tomography angiography. Other abbreviations are the same as those in Table 2.


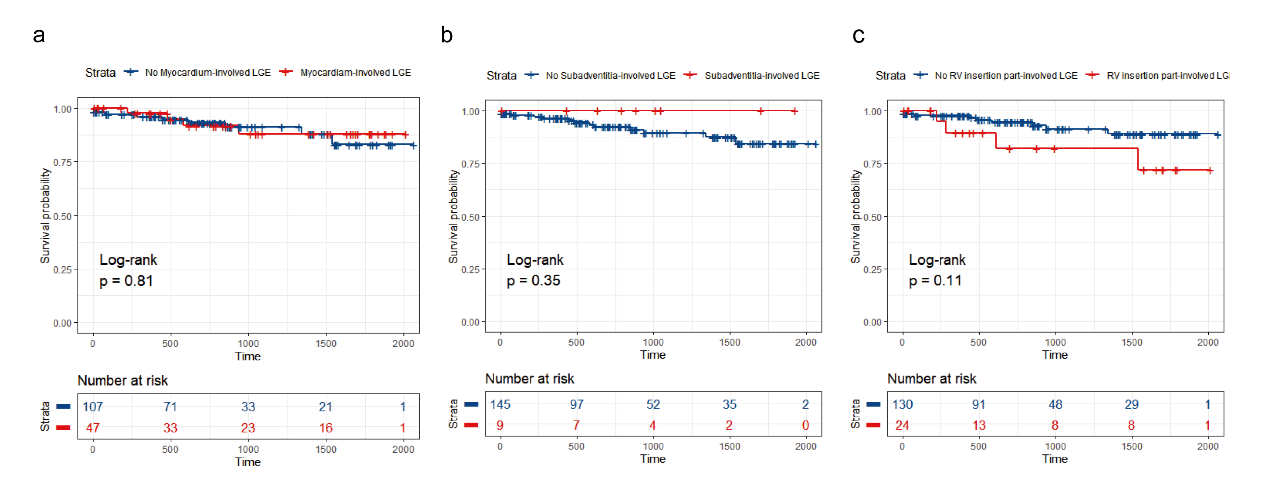


SUPPLEMENTAL FIGURE 6 Kaplan‒Meier Curves for Composite Events in All Patients after Excluding 22 Patients with LV Subendocardium-involved LGE

Curves for composite event-free survival are stratified by LV myocardium involvement (a), LV subadventitia involvement (b), and RV insertion points involvement (c) in patients without LV subendocardium-involved LGE. Abbreviations are the same as those in Table 2.
